# fMRI Repetition Suppression During Generalized Social Categorization

**DOI:** 10.1038/s41598-017-04115-8

**Published:** 2017-06-27

**Authors:** Tatiana Lau, Mina Cikara

**Affiliations:** Department of Psychology, Harvard University, 33 Kirkland Street, Cambridge, MA 02138 USA

## Abstract

Correctly identifying friends and foes is integral to successful group living. Here, we use repetition suppression to examine the neural circuitry underlying generalized group categorization—the process of categorizing in-group and out-group members across multiple social categories. Participants assigned to an arbitrary team (i.e., Eagles or Rattlers) underwent fMRI while categorizing political and arbitrary in-group and out-group members. We found that frontoparietal control network exhibited repetition suppression in response to “identical in-group” (Democrat-Democrat or Eagles-Eagles) and “different in-group” (Eagles-Democrat or Democrat-Eagles) trials relative to “out-group/in-group trials” (Republican-Democrat or Rattler-Eagles). Specifically, the repetition suppression contrast map included bilateral superior parietal lobule, bilateral dorsolateral prefrontal cortex (DLPFC), and bilateral middle temporal gyrus. Participants who reported an increased tendency to join and value their social groups exhibited decreased repetition suppression in bilateral DLPFC. Comparison of our whole-brain repetition suppression map with an independently identified map of frontoparietal control network revealed 34.3% overlap. Social categorization requires recognizing both a target’s group membership but also the target’s orientation toward one’s self. Fittingly, we find that generalized social categorization engages a network that acts as a functional bridge between dorsal attentional (exogenously-oriented) and default mode (internally-oriented) networks.

## Introduction

Group living confers significant advantages. By coordinating and cooperating with fellow in-group members, we reap numerous material and psychological benefits^1–5^. Social categorization—categorizing people into their respective social groups along a given dimension^6^—is critical for the successful navigation of group life. It is perhaps not surprising then, that there is a growing literature examining the neural basis of social categorization. These previous investigations have revealed a great deal about which brain regions and networks respond more to specific in-group versus out-group targets; however, most of these studies examine single groups or categories (in many cases marked by visual cues to group membership), which makes it difficult to determine whether these findings are unique to the categories under investigation or whether they reveal something more fundamental about the cognitive processes supporting social categorization.

The goal of the current investigation is to examine the neural circuitry underlying generalized in-group categorization—the process of identifying “us” across multiple social groups. Specifically, we use a repetition suppression paradigm to identify the neural responses associated with distinguishing in-group members from out-group members, independent of the features associated with the particular categories by which group boundaries are instantiated.

### Social categorization: More than mere similarity to the self

Social psychologists have long recognized that social categorization—the process of categorizing targets into their respective social groups^6^—is a separate process from self-categorization and social identification—the processes by which people categorize themselves and come to identify with specific social groups^7, 8^. Moreover, both of these are distinct from one potential consequence of these processes, namely evaluative bias, or in-group preferences^1, 9, 10^.

Previous fMRI studies of social categorization have primarily examined categorization across racial group boundaries. Several regions have been reliably associated with categorizing racial in-group and out-group members, including (but not limited to) anterior cingulate cortex, dorsolateral prefrontal cortex, medial prefrontal cortex, fusiform gyrus, and bilateral amygdala^11–13^. These regions are theorized to support processes ranging from differences in representing targets’ faces and motivational salience to perceivers’ mentalizing and emotion regulation. The broad circuitry recruited during race-based social categorization reflects, in part, the fact that categorization and identification are intimately intertwined—we do not just sort people into categories, we sort them into in-groups and out-groups, which are *egocentrically* defined.

Social categorization thus differs from other forms of categorization (e.g., sorting fruits versus vegetables) in that it may also spontaneously recruit representations of one’s own group membership (and associated preferences)^7, 8, 14^. Social identification is also a very flexible and dynamic process. Which specific social identity becomes salient in any given moment is highly context-dependent^14–17^. Thus, one process by which people may determine whether someone is an in-group member is via judgments of similarity to one’s self on some feature that is relevant to the current context (e.g., skin tone, nation of origin, display of symbols signaling religious or sports team affiliation).

Accordingly, several neuroimaging studies have attempted to identify an overlap between the brain regions implicated in self-referential processes and the categorization of in-group members. A relatively ventral area of medial prefrontal cortex (vmPFC), including pregenual anterior cingulate cortex (pgACC), is reliably associated with thinking about one’s own, as well as similar others’, traits, mental states, and characteristics^18–27^. Consideration of close others (e.g., family, a group of close friends, etc.) similarly elicits activation in these areas^28^. If “we” is represented similarly to “I”, this same region should exhibit greater activation in response to presentations of in-group relative to out-group targets.

Indeed, previous investigations have implicated the vmPFC/pgACC in the social categorization process, suggesting that some comparison to self or models of the self is inherent in the process of categorizing others. One experiment employing minimal groups reported that participants who were more biased in a resource allocation task (i.e., awarded more points to in-group than out-group players) exhibited greater mPFC activity relative to those who allocated resources equitably^29, 30^. Note, however, this specific region of activation was more dorsal than the regions typically associated with self-referential processes. Two other experiments in which participants categorized minimal groups (e.g., “Red Team”^31^) or words describing different social groups (e.g., “Australian”^32^) as “My Team” or “Other Team” also reported greater mPFC for in-group relative to out-group trials. In the minimal groups experiment, the region of activation was again more dorsal than the region associated with self-referential processing. The mPFC cluster in the real social groups experiment, by contrast, included both vmPFC and pgACC^32^. Closer inspection of the design of this experiment, however, suggests one should interpret these results with caution. The words that participants sorted as in-group or out-group labels were adjectives rather than social categories (e.g., “Australian, male” rather than “Australians, men”). Given that vmPFC/pgACC responses are higher for trait descriptions that are true versus false of the participant^33^, and higher for self-relevant facts and words (e.g., the participants’ own name) compared to irrelevant ones^34^, it is possible this pattern of activation is the result of making self-related attributes salient. The claim that mPFC/pgACC is associated with self-referential thought (including self-categorization) is uncontroversial. Less clear, however, is the extent to which self and similarity-driven processes are necessary or sufficient for categorizing others as in-group members.

Classic social psychological theories of intergroup relations remind us that in addition to similarity there are several other dimensions by which groups are defined, most notably common fate within groups^35^ and functional relations between groups^36^. In other words, groups are not only defined by the attributes that their members share; people also have strong expectations about the nature of the interactions and the obligations within and between groups^37^. Common fate—when individual group members’ outcomes are interdependent—is a critical cue for group boundary definition, and therefore social categorization. It increases perceptions of group cohesion within groups^38^ and promotes greater intergroup bias and discrimination between groups^39^. For example, when group member similarity, proximity, and common fate are independently manipulated, common fate is the only significant predictor of competitive, group-based aggression in the prisoner’s dilemma game^40^.

Abstracted to the group level, functional relations between groups—whether groups are cooperative, competitive, or independent—also determine who gets marked as friend or foe^41^. For example, cooperation between groups may (temporarily) change representations of out-group members to super-ordinate in-group members^41, 42^. Thus, rather than relying on an analysis that prioritizes similarity to oneself, another process through which people may categorize others as in-group members is by inferring the functional relations between one’s self and the target (e.g., “Are you with me or against me?”)^43^.

Very few neuroimaging studies have documented the brain regions and networks associated with tracking judgments of cooperation versus competition (and all of which are focused on interpersonal rather than intergroup dynamics). For example, in one experiment, playing a game with another person in both cooperative and competitive contexts (relative to playing alone) recruited the frontoparietal control network (FPCN) and anterior insula, which the authors speculated is related to greater attentional and executive demands required by tracking one’s own moves in relation to another’s^44^. Therefore rather than relying on vMPFC/pgACC, it is possible that generalized social categorization tracks targets’ functional significance (e.g., good or bad for me?), and therefore draws on domain-general circuitry associated with goal-directed information integration^45, 46^.

### Increasing the generalizability of inferences about the neural basis of social categorization

Though neuroimaging investigations have revealed a great deal about what regions of the brain encode race^11–13^, gender, and a variety of other significant social categories^47^, we know comparatively little about generalized social categorization: how we distinguish between “us” and “them” more broadly. It is imprudent to make inferences about generalized group processes from investigations of single social groups (especially those marked by visual cues to group membership) because they are intrinsically confounded with differences in the visual appearance of targets, associated stereotypes and prejudices, and perceivers’ familiarity with the groups in question. For example, the neural correlates of race-based categorization could theoretically overlap with the network supporting generalized social categorization processes, but also with circuitry representing cultural stereotype knowledge, episodic memory, and other processes uniquely associated with race. In this example, there is no way to parse which components are common across categories versus specific to race. Even experiments demonstrating overlapping activation across multiple social categories are limited so long as they employ a traditional univariate analysis approach. Given the limited spatial resolution of fMRI, overlapping activation within a region across categories (e.g., in-group members from multiple categories) may arise from averaging across neighboring but distinct subpopulations of neurons.

Repetition suppression paradigms circumvent these technical limitations and enable stronger inferences about the common neuronal populations supporting the representation of shared properties across distinct stimuli. Repetition suppression refers to the well-documented phenomena that stimulus-, property-, or concept-tuned neurons respond less upon repeated exposure to their preferred (as compared to irrelevant) inputs^48^. For example, if researchers are interested in identifying brain regions that not only represent faces, but specific identities, they may expose participants to two conditions: one in which an identical face is presented two times in a row and another in which one face is followed by another face of a different identity. Regions (or populations of neurons) that are specifically tuned to identity representations will exhibit repetition suppression in the former but not the latter case despite the fact that a face is always followed by another face. If these regions are truly identity-tuned they should similarly show suppression if the same identity is repeated but changes on a different dimension (e.g., same face exhibiting happy followed by neutral emotional expression^49^). As such, researchers have now leveraged repetition-related reductions in fMRI BOLD responses to characterize the functional properties of different brain regions at sub-voxel resolution across a wide variety of tasks and processes, including, but not limited to, visual^50, 51^ and language processing^52, 53^, action representation^54^, trait inference^21, 55^, and mentalizing^23^.

Thus by many accounts, fMRI repetition suppression in response to repeated but distinct stimuli indicates that both stimuli share some property or cognitive process that engages the same underlying neuronal population. For our purposes, these stimuli must be similar on only one rather than several dimensions—in-group status—for the results to be maximally informative. For example, if a liberal Bostonian exhibited repetition suppression in region X upon exposure to the second in a pair of Red Sox fans, one could not distinguish whether the neurons in region X are sensitive to the second target’s in-group status, their association with baseball, or any other feature the two targets share. If, however, the liberal Bostonian was first exposed to a Red Sox fan followed by a fellow Democrat, and exhibited repetition suppression in region X after viewing the Democrat target, the space of reasonable inferences about region X’s preferred stimulus class narrows (e.g., in-group status, familiarity).

The current experiment builds on and extends past research on the neural basis of social categorization in three important ways. First, we employ multiple, objectively orthogonal social categories to test the generalizability of our results across group boundaries. Second, we include both real world (i.e., political parties) and novel groups (i.e., arbitrary teams) to ensure the generalizability of our results is not driven by stereotype content, familiarity, or other properties shared among real-world social categories. Finally, we employ a repetition suppression paradigm to avoid the problems associated with interpreting overlapping mean activations in our fMRI data.

### Overview and Hypotheses

**Figure 1:**
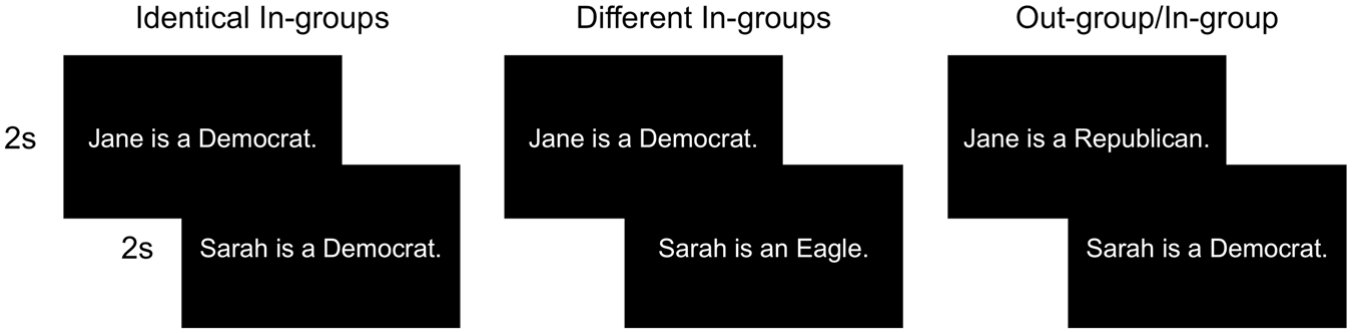
Task Stimuli. Trials consisted of pairs of statements, presented sequentially, in white font against a black background. The experiment included 3 condition types: (**a**) identical in-groups, (**b**) different in-groups, or (**c**) out-group/in-group. Each pair was followed by a 2s prompt that asked them, “How many of the people described were members of your in-group?”

In order to examine the neural circuitry underlying generalized in-group categorization, we assigned participants to an arbitrary team (i.e., Eagles or Rattlers) and recorded their BOLD responses while they categorized political and arbitrary in-group and out-group members. Following standard repetition suppression paradigms, each trial included two targets, representing one of three combinations: “identical in-group” (Democrat-Democrat or Eagles-Eagles), “different in-group” (Eagles-Democrat or Democrat-Eagles), and “out-group/in-group trials” (Republican-Democrat or Rattler-Eagles; see Fig. 1). In-group categorization sensitive regions should exhibit reduced activation during identical in-group and different in-group trials relative to out-group/in-group trials. Following past findings, if generalized in-group categorization relies on self-referential processes, this analysis should identify a network that includes vmPFC/pgACC. If instead in-group categorization relies on an analysis of functional relations between the target and oneself (e.g., “Is the target a friend or foe?”), then our analysis should identify the FPCN (previously associated with encoding functional relations) or an alternative network. All experiment materials, summary data, and analysis code can be downloaded at: https://osf.io/epnv6.

## Results

### Behavioral Results

Participants reported more positive evaluations of their arbitrary team, the Eagles (*M* = 63.98, *SD* = 15.98) relative to their competitors, the Rattlers (*M* = 33.52, *SD* = 16.05), mean difference = 30.47, 95% CI [19.63, 41.31], *t*(21) = 5.85, *p* < 0.001, *d* = 1.902. Categorization accuracy did not differ by condition, *F*(2, 42) = 0.581, *p* > 0.250, *d* = 0.004. Reaction times, however, did. A repeated-measures ANOVA revealed a main effect of condition, *F*(2,42) = 3.799, *p* = 0.03, generalized eta-squared = 0.019: pairwise comparisons revealed reaction times were significantly shorter in the identical in-group condition (*M* = 0.72 s, *SD* = 0.16) relative to the out-group/in-group condition (*M* = 0.67 s, *SD* = 0.15), mean difference = 0.052 s, 95% CI [0.007, 0.097], *p* = 0.020, *d* = 0.253. Reaction times in the different in-group condition (*M* = 0.69 s, *SD* = 0.15) did not differ from the other two conditions (*ps* > 0.213). Despite the condition differences, this specific pattern of response times suggests our fMRI contrast effects cannot be accounted for by condition differences in effort or task difficulty.

### fMRI Results

#### Analysis of identical versus different in-group trials

We first conducted a whole-brain contrast to determine which, if any regions, exhibited differences in repetition suppression for identical versus different in-group trials. No clusters survived correction for multiple comparisons, so we moved forward with the main repetition suppression analysis of generalized social categorization collapsing across both in-group/in-group conditions and comparing them to the out-group/in-group condition.

#### Repetition suppression network for in-group targets

The whole brain analysis of repetition suppression for in-group targets (Out-group/In-group > Identical In-group and Different In-group) identified the frontoparietal control network, including bilateral superior parietal lobule, bilateral dorsolateral prefrontal cortex, and bilateral middle temporal gyrus (Table 1).

**Table 1:** Out-group/In-group > (Identical In-group, Different In-group): Repetition Suppression.

Region	L/R	*x*	*y*	*z*	Cluster Size (Voxels)
Superior Parietal Lobule	R	40	−58	48	1457
Superior Parietal Lobule	L	−40	−54	54	2851
Dorsolateral Prefrontal Cortex	R	48	34	38	544
Dorsolateral Prefrontal Cortex	L	−44	30	28	1041
Middle Temporal Gyrus	R	64	−28	−12	483
Middle Temporal Gyrus	L	−58	−40	−12	472

Coordinates refer to peak voxel in Montreal Neurological Institute stereotaxic space.

To confirm that our results reflected repetition suppression rather than different responses to the first statement (e.g., greater responses to out-group relative to in-group sentences, which could also drive the Out-group/In-group >(Identical In-group, Different In-group) contrast) we extracted percent signal change (PSCs) for reach ROI for the repetition time window (TR) corresponding to the presentation of the first sentence and the TR corresponding to the presentation of the second sentence. Because there was no jittered ITI between our first and second stimuli in each trial, these events could not be modeled separately. We did not, however, find a significant difference across conditions in the PSC’s extracted during the first sentence of each trial in any of our six ROI’s (across all ROIS all *t*s(42) < 1.92, Holms adjusted *p*s > 0.37). Furthermore, PSCs from the second screen exhibited the predicted repetition suppression pattern: the highest response in the out-group/in-group condition, with significantly lower responses in the identical in-group and different in-group conditions, which were not different from each other (out-group/in-group > identical in-group, different in-group, across all ROIs, all *t*s(42) >2.68, Holms adjusted *p*s ≤ 0.01; see Supplementary Information for plots of PSC curves and t-statistics for each ROI listed in Table 1). Thus, if anything, our averaging approach skews conservative: the effect of repetition suppression is being underestimated by including the window corresponding to the first sentence (which again is not significantly different across conditions) in the event.

Interestingly, we found a negative correlation between the degree to which participants reported valuing and joining groups (calculated as the average of the 12 questions asked in the participant recruitment survey) and the degree to which bilateral DLPFC exhibited repetition suppression (calculated as the parameter estimate of the out-group/in-group condition minus the average of the parameter estimates of the identical in-group and different in-group conditions): *r*(20) = −0.38, *t*(20) = −1.87, *p* = 0.076 for left DLPFC; *r*(20) = −0.47, t(20) = −2.38, *p* = 0.027 for right DLPFC). In other words, participants who reported an increased tendency to join and value social groups exhibited a decreased repetition suppression effect. None of the other regions identified by the whole-brain repetition suppression contrast correlated with this group measure.

#### Overlap with independently identified frontoparietal control network

**Figure 2:**
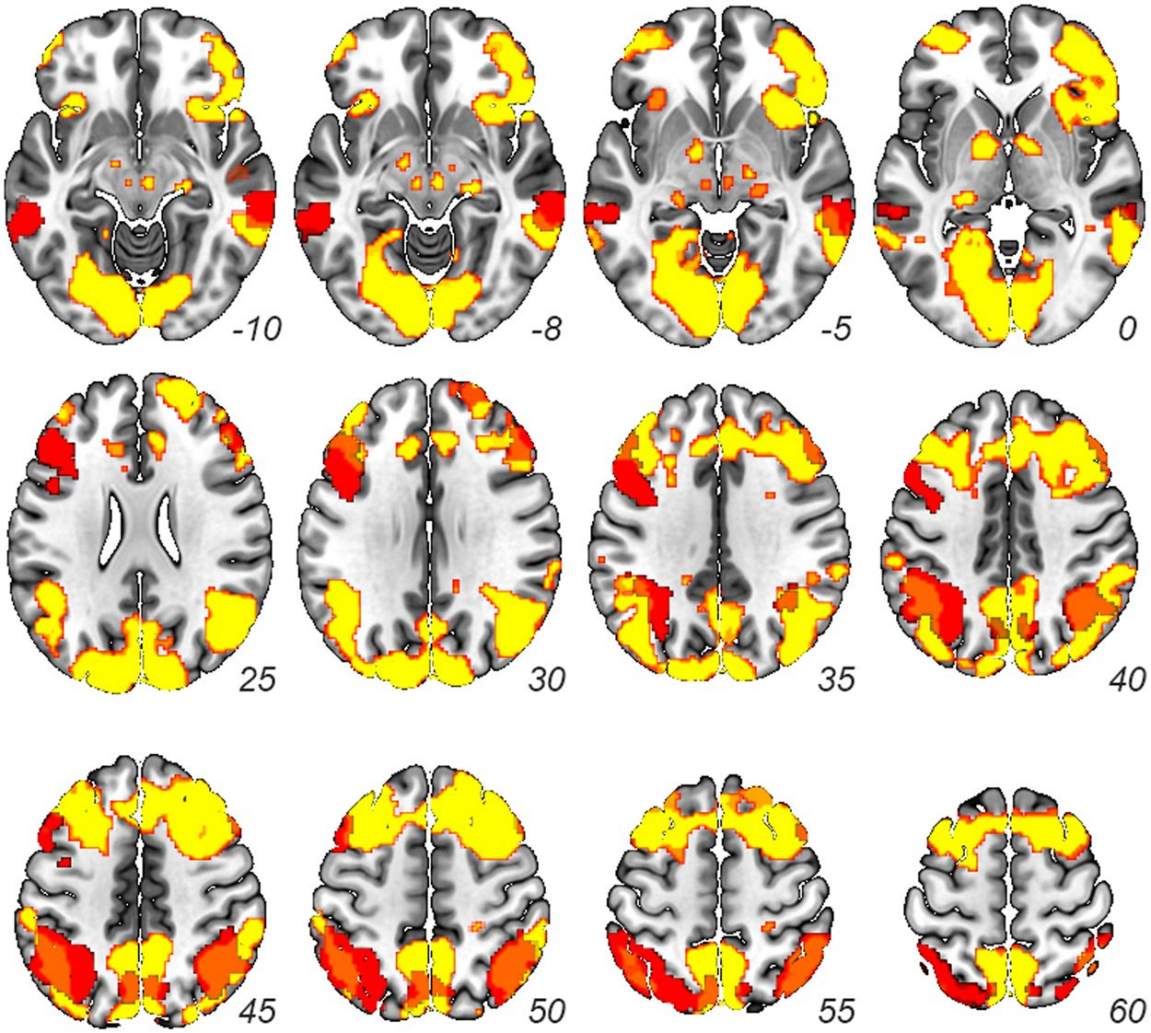
Repetition Suppression. Results from whole-brain contrast of out-group/in-group trials > identical in-group, different in-group trials (red; *p* < 0.001 corrected from *p* < 0.005) overlaid on top of resulting FPCN map resulting from (45) (yellow). Orange denotes overlap between the two maps.

We compared the degree of overlap between the network identified by our repetition suppression analysis and a network map generated by (45) based on the partial least squares analysis of connectivity between frontoparietal control network and default and dorsal attentional networks, respectively. The degree of overlap, calculated as the number of voxels that overlapped between the two functional maps (2348 voxels) divided by the number of voxels in our whole-brain results (6848 voxels), was 34.29% (see Fig. 2).

#### Repetition Enhancement for in-group targets

**Figure 3:**
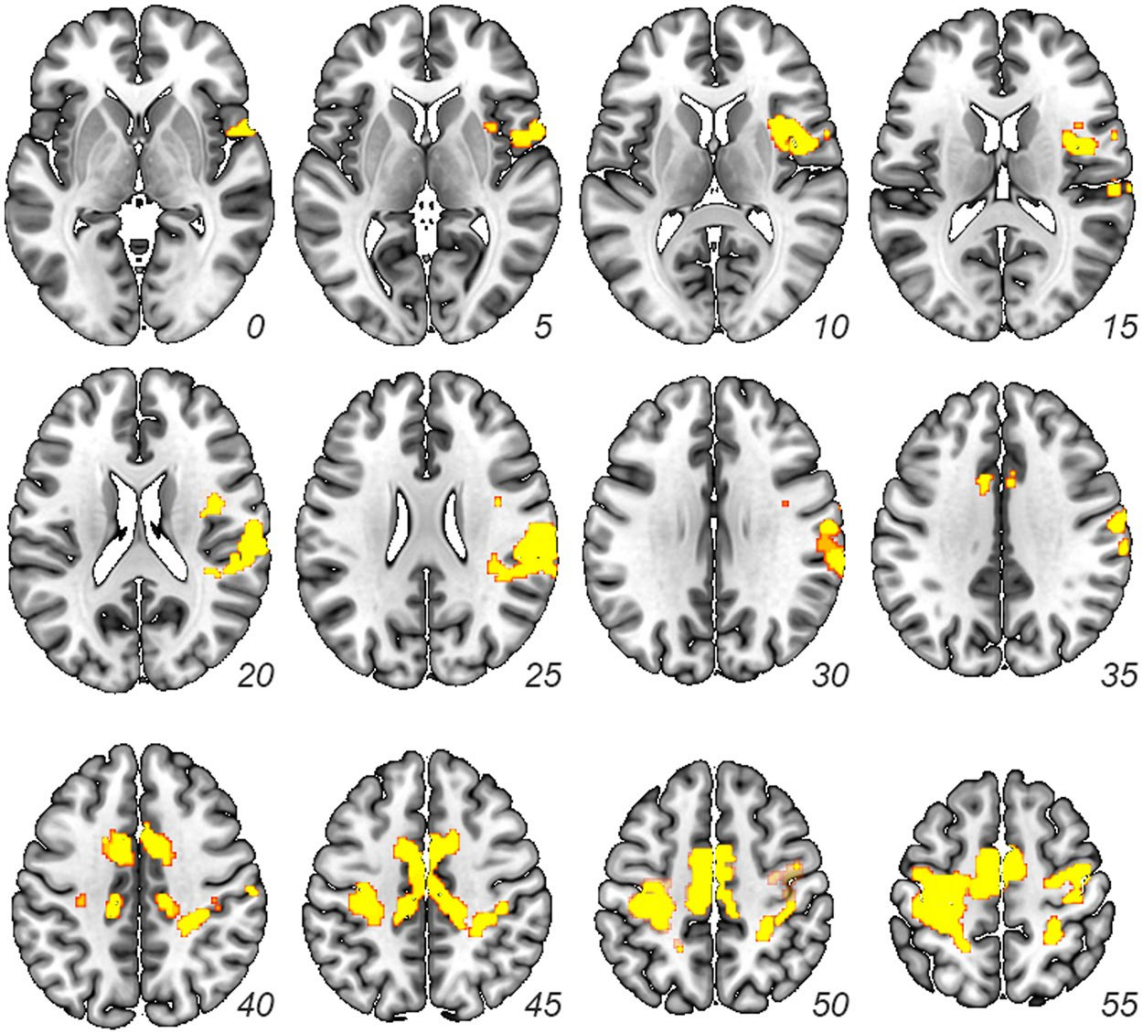
Repetition Enhancement. Results from whole-brain contrast of identical in-group, different in-group > out-group/in-group trials (*p* < 0.001 corrected from *p* < 0.005).

Though we designed our experiment to analyze repetition suppression, we also assessed whether any regions exhibited repetition enhancement for in-group targets. While repetition enhancement effects are not as well understood, they are generally taken as a proxy of greater processing of stimuli^56^. The whole-brain repetition enhancement contrast (Identical In-group and Different In-group > Out-group/In-group) identified the network of regions including right temporoparietal junction and insula (see Table 2 and Fig. 3).

**Table 2:** (Identical In-group and Different In-group) > Out-group/In-group: Repetition Enhancement.

Region	L/R	*x*	*y*	*z*	Cluster Size (Voxels)
Postcentral Gyrus	L	−42	−30	66	4217
Precentral Gyrus	R	36	−18	66	709
Supramarginal Gyrus/rTPJ	R	40	−38	24	682
Insula/Frontal Operculum	R	48	2	10	425

Coordinates refer to peak voxel in Montreal Neurological Institute stereotaxic space.

## Discussion

We used a repetition suppression paradigm to examine the neural substrates of the process of categorizing others as in-group members across multiple social categories. A network roughly corresponding to the FPCN exhibited repetition suppression in response to repeated in-group trials. Thus, in contrast to previous experiments, which have highlighted vmPFC/pgACC and self-referential processes in social categorization, the present findings indicate that the general process of categorizing “us” relies on domain-general circuitry associated with goal-directed information integration. Note, however, that many of these previous experiments were designed to elicit trait judgments, whereas our experiment was designed to engage categorization without necessarily engaging trait judgments. While we agree that group categorizations may inform trait judgments of individuals eventually, we argue that there is nothing about social categorization itself that requires it. Moreover, we found that the degree of repetition suppression in bilateral DLPFC correlated negatively with the degree to which participants reported valuing and joining groups.

The FPCN, first identified in a resting-state seed-based functional connectivity analyses^57^ and network parcellation analyses^58^, is a network independent from the dorsal attention network (DAN) and the default mode network (DMN). It includes several regions that have previously been associated with attentional control, working memory, decision-making, and information integration^59–65^. These early connectivity results led researchers to theorize that the FPCN acts as a functional bridge for networks that support externally directed attention and cognitive control.

More recent investigations of task-related functional connectivity with FPCN confirm that it couples flexibly with both DMN and DAN^66^. Specifically, FPCN activity does not correlate negatively with the DMN (as would a “task-positive” network); instead it correlates positively with *both* DMN and the DAN, depending on the task. While the DMN is reliably associated with self-referential processes (and prospection more generally^67^), the DAN is primarily associated with exogenously cued attention^68^. The FPCN, on the other hand, appears to plays a key role in the integration of goal-directed information over time, for both endogenously and exogenously oriented tasks. For example, the FPCN is coupled with the DMN during autobiographical planning but coupled with the DAN during visuospatial planning^45, 46^. Explicitly planning how one is going to execute a task elicits activation in the FPCN, whereas simulating the outcomes of the task does not^69^. And finally, integrating evidence from the external environment that disconfirms one’s priors also recruits the FPCN^70^.

Given its connectivity to the DMN and DAN, as well as its purported functional role in goal-directed information integration, the FPCN may be particularly well suited to service the process of categorizing others as in-group members. Our ability to correctly identify others as in-group or out-group members necessitates a comparison between the demands of the current environment and our internally generated representations of ourselves as members of a given group. While we may be in-group members with specific people in one setting (e.g., Boston Red Sox fans at a baseball game), we may not be in the same group in a different setting (e.g., residents of two Boston neighborhoods negotiating next year’s trash collection schedule), and our ability to correctly identify and categorize in-group members in different settings relies on the ability to precisely reconcile the environment at hand with our self-categorization. Whether we share a common fate with another person is dictated by the social context; thus, a social categorization network would need to be able to flexibly integrate environmental cues with salient self-related knowledge.

We would be remiss if we did not highlight that there is considerable variability in the extent to which the three sets of regions exhibiting repetition suppression in the present study overlap with the FPCN identified by (45). Specifically, our bilateral DLPFC and MTG results overlap less with this map than bilateral SPL (Fig. 2). Given that the two networks were defined using completely different tasks and analyses, this variability is to be expected. Though this is purely speculative, one possibility is that different nodes of the FPCN may be associated with distinct sub-processes (e.g., similarity and functional relation judgments) that support social categorization (as well as other non-social processes). For example, the DLPFC, wherein we found a negative correlation between the degree of repetition suppression and the self-reported propensity to value and join groups, may be critical for focusing our attention on salient cues to targets’ group membership (and inhibiting less immediately relevant information). Those who are more group-oriented may pay equal attention to in-group and competitive out-group targets, reducing the repetition suppression effect between conditions. DLPFC activity, in turn, may correlate with DMN versus DAN depending on which dimensions participants emphasize when categorizing others. SPL, in contrast, may be more closely associated with encoding contextual information (e.g., “Am I making a judgment of group membership through a political party or arbitrary team lens?”). This experiment is merely a first step in better understanding the extent to which generalized social categorization relies on domain-general circuitry.

The whole-brain repetition enhancement contrast (i.e., Identical In-group and Different In-group > Out-group/In-group) revealed that in-group targets spontaneously engaged rTPJ and insula to a greater degree than out-group targets did. These findings comport with previous experiments indicating that in-group targets are more likely to engage brain regions associated with representing motivational salience^71, 72^ and theory of mind^73^, operations which are reliably associated with insula and rTPJ engagement, respectively.

Though the current results speak to the neural substrates of generalized social categorization as a process, they do not address which feature or dimension participants are using to distinguish in-group and out-group targets across multiple category boundaries. Valence, specifically functional significance or evaluation, is a likely candidate for the dimension distinguishing representations of ‘us’ and ‘them’ across multiple categories. This account is consistent with decades of theorizing that emphasizes the priority of functional relations as an organizing principle for group-related perception and cognition^8, 35, 41, 74^.

The present findings also do not clearly adjudicate between a similarity versus functional relation account of social categorization (presumably because categorization relies on both judgments). Future experiments could manipulate which strategies people employ to categorize others: for example, we could instruct participants to make similarity or functional relation judgments on different social categorization trials. We predict that FPCN would couple with the DMN (including mPFC) more strongly on similarity judgment trials, but more strongly with DAN on functional relation judgment trials. Furthermore, degree of connectivity among networks may vary based on the types of social groups under consideration. For example, we might observe greater DMN/FPCN coupling when the environment prioritizes categorization along a static group boundary (e.g., race) because similarity is a reliable indicator of group membership, whereas we might observe greater DAN/FPCN coupling when people categorize along dynamic, functional group boundaries (e.g., common fate irrespective of other group category cues). These experiments and similar approaches may help reconcile seemingly discrepant findings in the literature and provide a stronger foundation for future research.

In using a repetition suppression paradigm to determine the areas responsible for general in-group categorization, we have shown evidence that the process of generalized in-group categorization relies not solely on regions associated with self-referential processes but on a network that can flexibly couple with networks associated with self-referential and external attention orientation processes. Because we so readily categorize in-group members and because these categorizations drive how we treat one another (e.g., favoring in-group members at the expense of out-group members), both the neuroscience and psychology literatures on intergroup dynamics benefit from a deeper understanding of how this coupling works in service of broad group categorization.

## Methods

### Participants

We recruited twenty-three, right-handed, native English speakers (12 female, *M*_age_ = 25.58 years, *SD* = 3.12 years) from the community based on their responses to a larger online survey (see Participant Selection below). All participants self-identified as Democrats. One participant was excluded from analysis because of excessive head movement (greater than 2mm) while in the scanner. Thus, the final pool of participants comprised of 22 people (12 female, *M*_age_ = 25.25 years, *SD* = 2.77 years). Carnegie Mellon University’s IRB committee approved all experimental procedures; methods were carried out in accordance with the relevant guidelines and regulations, and we obtained informed consent from each participant.

#### Participant selection

Survey participants (N = 745) were recruited to participate in an online problem-solving challenge for a chance to win a $30 gift card. For the purposes of the challenge, participants were assigned to one of two teams (the Eagles and the Rattlers), ostensibly based on their answers to five personality items. In reality, all participants were assigned to the same team, the Eagles^1^. Following team assignment, participants answered 12 questions assessing their propensity to value and join groups (e.g., “The social groups we belong to are one of the most important things in our lives” and “We are defined, at least in part, by the social groups that we belong to”; *Strongly Disagree* (1) to *Strongly Agree* (7), Cronbach’s α = 0.73 for the final pool of participants). Participants were then asked for demographic information (age and gender) and their political party affiliation (or lack thereof) and asked to rate how much they liked, valued and felt connected to their party on three 100-point scales that ranged from *Not at All* (0) to *Extremely* (100). Survey participants could then include their e-mail addresses if they were interested in participating in a related fMRI study. They were also asked to confirm or disconfirm a series of statements relating qualifications for participating in an fMRI study (e.g., whether they had metal in their body, being able to lie still for over an hour, etc.). Following this, participants were informed that enough data had been collected for the time being and that they would not need to complete a problem-solving challenge at that time.

Respondents interested in the fMRI study were then invited to participate if they reported no contra-indicators and reported that they liked and valued the Democratic Party in excess of the midpoint of the scale (for the two items, Cronbach’s α = 0.86).

### Procedure

#### Pre-scanning tasks

Prior to being scanned, participants reported whether or not they recalled their team assignment; all but two participants remembered their team assignment. We also showed participants a social network diagram illustrating that they were much more similar to their teammates (and that the competing players were much more similar to one another) than the groups were to each other (increased group cohesion increases intergroup bias; manipulation is identical to that found in Experiment 4 in (ref. 75)). We explained that the participants’ own team had accumulated 82 points whereas the other team had earned 84 points indicating that it was a tight race. Whichever team had the higher score at the end of the experiment would win a bonus of $10. Participants then rated how they much they liked, valued, and felt connected to the Eagles and the Rattlers on three 100-point scales that ranged from *Not at All* (0) to *Extremely* (100; Cronbach’s α’s = 0.82 and 0.76 for the Eagles and Rattlers, respectively). Finally, participants completed a series of practice trials in preparation for the main task.

#### Main Task

After being placed in the scanner, participants performed two runs of a task for a separate group evaluation experiment. On each trial in this evaluation task, participants saw a single sentence describing a person’s category membership (e.g., “Sam is Democrat”). After the statement disappeared, participants pressed one button to indicate they felt positively toward the target or another button to indicate they did not. If anything, this intermediate task allowed participants to acclimate to the scanner environment and primed group membership as a dimension of interest for the main repetition suppression experiment. The evaluation experiment will not be discussed further here.

Participants then began the main repetition suppression experiment. In each trial, participants read paired statements about two targets (see Fig. 1). Targets were members of (a) the Eagles (b) the Rattlers, (c) the Democratic Party, or (d) the Republican Party. Following standard repetition suppression paradigms, statements in each pair described people belonging to one of three conditions: ‘identical in-group’ (Democrat-Democrat or Eagles-Eagles), ‘different in-group’ (Eagles-Democrat or Democrat-Eagles), and ‘out-group/in-group trials’ (Republican-Democrat or Rattler-Eagles). All target names were gender-matched to the participant. Within each trial, each description statement was shown for 2s, followed by a 2s prompt, during which the participants answered, “How many of the people described were members of your in-group?” (neither/one/two). Each trial was followed by a fixation cross which lasted 4s–16s (jittered). Participants saw 8 trials of each condition type in each run. Condition order and trial timing were optimized using the optseq algorithm (http://www.surfer.nmr.mgh.harvard.edu/optseq). Participants completed 8 runs total, approximately 6 minutes each.

### fMRI data acquisition, preprocessing, and analysis

We collected data using a 32-channel head coil in a 3.0-tesla Verio MRI scanner (Siemens) at the Scientific Imaging & Brain Research Center at Carnegie Mellon University. At the beginning of each scan session, we acquired a high-resolution T-1 weighted anatomical image (T1-MPRAGE, 1 × 1 × 1 mm, parallel to the anterior commissure-posterior commissure plane) for use in registering activity to each participant’s anatomy and spatially normalizing data across participants. Functional images were then acquired through eight echo-planar imaging (EPI) sessions lasting six minutes on average. For near whole brain coverage, we acquired 36 interleaved 3.0mm slices (repetition time = 2s; echo time = 29 ms; flip angle = 79 degrees; field of view = 192 mm; matrix = 64 × 64).

We conducted preprocessing and statistical analyses using SPM8 (Wellcome Trust Centre for Neuroimaging, London, UK, http://www.fil.ion.ucl.ac.uk/spm). We realigned functional images to the first volume, co-registered images to the individual’s anatomical scan, and normalized images to a standard EPI template using a Montreal Neurological Institute (MNI) reference brain, resliced to 2 mm × 2 mm × 2 mm voxels, and smoothed using a 5 mm FWHM Gaussian kernel.

We modeled data with an event-related design using a general linear model. For each of the eight runs, four regressors— three condition regressors (i.e., the first four seconds of each trial), and one regressor modeling all decision periods (i.e., the final two seconds)—were convolved with the canonical hemodynamic response function. In addition, we included nuisance regressors containing the temporal and spatial derivatives for each of the main regressors and eight run regressors. We then entered the resulting contrast images into a second-level analysis that treated participants as a random effect. We applied the contrast, [out-group/in-group > (identical in-group, different in-group)], to the entire brain in order to identify regions exhibiting repetition suppression associated with in-group categorization. To reduce the number of comparisons across the whole brain, we generated a mask using FSL’s MNI structural atlas that masked out the cerebellum, brain stem, ventricles, occipital lobe, and white matter. We chose to exclude occipital lobe because the stimuli were text-based and we controlled for the number of characters in each statement string across conditions. A Monte Carlo simulation—AFNI’s 3dClustSim (http://afni.nimh.nih.gov/pub/dist/doc/program_help/3dClustSim.html)—determined a minimum cluster size of 365 voxels to achieve corrected p < 0.001 whole-brain contrasts, with a voxelwise threshold of p < 0.005. Note that a cluster-defining threshold of p < 0.001 with the updated 3dClustSim function has a false positive of only 8.6%^76^.

## Electronic supplementary material

Supplementary Information
