# Supplementary material for: fMRI Repetition Suppression During Generalized Social Categorization
Source: Sci Rep. 2017 Jun 27;7:4262. doi: 10.1038/s41598-017-04115-8 (PMC5487342; doi:10.1038/s41598-017-04115-8)

**Supplemental Materials for**

**“fMRI Repetition Suppression During Generalized Social Categorization”**

T-tests comparing out-group/ingroup > (identical in-group, different in-group) PSCs by ROI in TR corresponding to first statement screen (3^rd^ TR after onset):

Right SPL: *t*(42) = 0.018, *p* = 0.99; adjusted *p* = 1.0000000

Left SPL: *t*(42) = 1.16, *p* = 0.25; adjusted *p* = 0.7603825

Right DLPFC: *t*(42) = 0.18, *p* = 0.86; adjusted *p* = 1.0000000

Left DLPFC: *t*(42) = 1.91, *p* = 0.06; adjusted *p* = 0.3677031

Right MTG: *t*(42) = 1.85, *p* = 0.07; adjusted *p* =  0.3677031

Left MTG: *t*(42) = 1.92, *p* = 0.06; adjusted *p* = 0.3677031

T-test comparing out-group/ingroup > (identical in-group, different in-group) PSCs by ROI in TR corresponding to second statement screen (4^th^ TR after onset):

Right SPL: *t*(42) = 3.16, *p* = 0.0029; adjusted *p* = 0.012

Left SPL: *t*(42) = 4.57, *p* = 0.0000; adjusted *p* = 0.00026

Right DLPFC: *t*(42) = 2.68, *p* = 0.010; adjusted *p* = 0.012

Left DLPFC: *t*(42) = 4.02, *p* = 0.0002; adjusted *p* = 0.0012

Right MTG: *t*(42) = 3.15, *p* = 0.0030; adjusted *p* = 0.012

Left MTG: *t*(42) = 3.13, *p* = 0.0031; adjusted *p* = 0.012


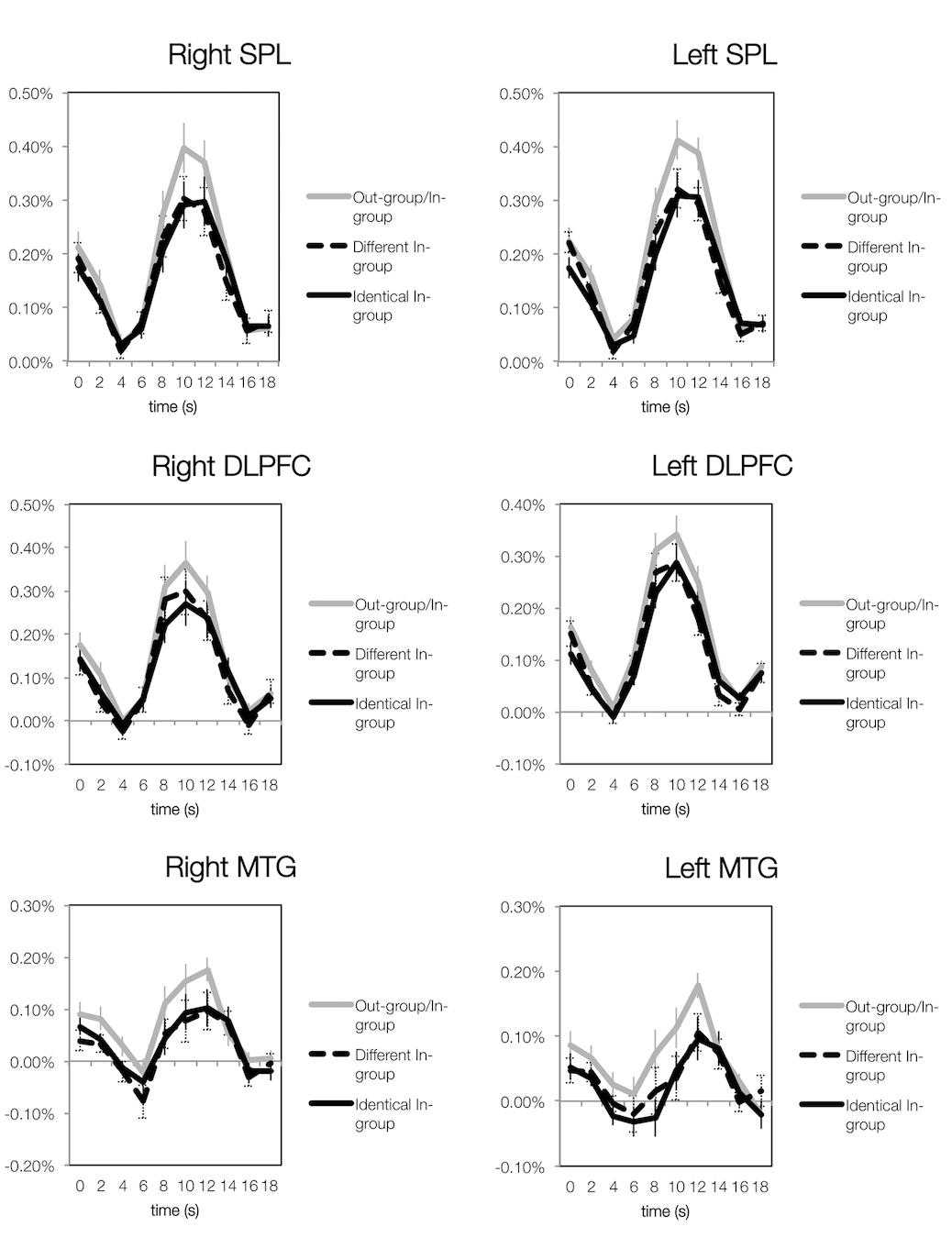

Supplement: Supplementary file 1 — Supplementary Information [file 41598_2017_4115_MOESM1_ESM.doc]
